# Economic Evaluation in Stratified Medicine: Methodological Issues and Challenges

**DOI:** 10.3389/fphar.2016.00113

**Published:** 2016-05-09

**Authors:** Hans-Joerg Fugel, Mark Nuijten, Maarten Postma, Ken Redekop

**Affiliations:** ^1^Department of Pharmacy, University of GronigenGroningen, Netherlands; ^2^ARS Accessus MedicaAmsterdam, Netherlands; ^3^Institute of Health Policy & Management, Erasmus University RotterdamRotterdam, Netherlands

**Keywords:** stratified medicine, health technology assessments, guidelines, reimbursement mechanisms, reimbursement, biomarkers

## Abstract

**Background:** Stratified Medicine (SM) is becoming a practical reality with the targeting of medicines by using a biomarker or genetic-based diagnostic to identify the eligible patient sub-population. Like any healthcare intervention, SM interventions have costs and consequences that must be considered by reimbursement authorities with limited resources. Methodological standards and guidelines exist for economic evaluations in clinical pharmacology and are an important component for health technology assessments (HTAs) in many countries. However, these guidelines have initially been developed for traditional pharmaceuticals and not for complex interventions with multiple components. This raises the issue as to whether these guidelines are adequate to SM interventions or whether new specific guidance and methodology is needed to avoid inconsistencies and contradictory findings when assessing economic value in SM.

**Objective:** This article describes specific methodological challenges when conducting health economic (HE) evaluations for SM interventions and outlines potential modifications necessary to existing evaluation guidelines /principles that would promote consistent economic evaluations for SM.

**Results/Conclusions:** Specific methodological aspects for SM comprise considerations on the choice of comparator, measuring effectiveness and outcomes, appropriate modeling structure and the scope of sensitivity analyses. Although current HE methodology can be applied for SM, greater complexity requires further methodology development and modifications in the guidelines.

## Introduction

The concept of “Stratified Medicine”(SM) is becoming a practical reality with the targeting of medicines by using a biomarker or genetic-based diagnostic to identify the eligible patient sub-population (Payne and Annemans, 2013). The quantity of biomarkers, prognostic, and diagnostic tests available for patients has increased significantly over the last decade and SM interventions are increasingly being developed and used in clinical care. In the SM concept, subgroups of responders are selected or identified based on risk of disease or response to therapy, with the notion to improve treatment outcomes in these subgroups by increasing efficacy and/or reducing toxicity. This stratification of the population by using diagnostic tests or techniques is intended to reduce the use of ineffective or unsafe drugs, which should translate into improved health outcomes for patients and more efficient use of health care resources. However, there is much debate and uncertainty on which SM tests provide economic value and how to balance the need for innovative new technologies with affordability. Decision makers and stakeholders need information on which tests provide added value in order to make appropriate decisions about where to invest efforts in development and adoption (Phillips et al., 2014). A number of analysts have observed that the promise of SM is yet to be realized, partly due to the lack of sufficiently robust clinical and economic evidence based to support the widespread use in clinical practice (Faulkner et al., 2012; Berger and Olson, 2013; The Academy of Medical Sciences, 2013; Phillips et al., 2014; Rogowski et al., 2014). Several published systematic reviews had suggested there are limitations in the quantity and quality of economic evaluations of examples of targeted therapies, imposed by weak clinical and economic evidence base (Vegter et al., 2010; Wong et al., 2010; Hatz et al., 2014). Annemans et al. as well as Buchanan et al explored methodological challenges of conducting economic evaluations of targeted interventions, and outlined new measurement issues for traditional cost-effectiveness analysis (CEA) when adding a test or sequence of tests into the clinical car pathway (Annemans et al., 2013; Buchanan et al., 2013). Furthermore, there is uncertainty in methods to be used with testing of multiple biomarkers or clinical applications based on whole exome or genome sequencing*s*. In addition, challenges arise if the economic evaluation of SM interventions is understood as an evaluation of the benefits, harms and cost-effectiveness at the individual patient preference level; (Basu, 2011; Rogowski et al., 2014) it should rather be conceived as applying to subpopulations as a whole.

Methodological standards and guidelines exist for economic evaluations in clinical pharmacology and are an important component of programs for health technology assessment (HTAs) in many countries. However, these guidelines have initially been developed for traditional pharmaceuticals and not for complex interventions with multiple components. This raises the issue as to whether these standards and guidelines are adequate to address more targeted approaches to therapy or whether new specific guidance and methodology is needed to avoid inconsistencies and contradictory findings when assessing economic value in SM.

This article addresses key methodological issues and challenges when conducting health economic evaluations for SM interventions and outlines potential modifications necessary to existing evaluation guidelines and principles that would promote consistent economic evaluations for decision making in SM. Utilizing a set of criteria represented by the guidelines for cost-effectiveness (such as, ISPOR, NICE)^1^^,^^2^ we identified various aspects of the criteria/guidelines which require specific attention/modification for SM interventions.

## Specific methodological aspects in SM

While the basic framework for economic evaluations of SM interventions is similar to traditional clinical pharmacology some specific issues and challenges can be identified and assessed based on economic evaluation checklists^2^ (Huseruau et al., 2013; see Table 1).

**Table 1:** **Summary of methodological issues in economic evaluations of SM interventions**.

**Statements**	**Guidelines**	**Issues /challenges**	**Possible solutions/new guidance**
Perspectives and Target Audience	Societal or third party	Societal perspective is preferred (ideally), although third-party is most used. Funding silos may lead to different payer perspectives, i.e., those paying for drugs vs. those paying for diagnostics.	Clear understanding of target audience and further specification of what defines a third-party-payer will increase relevance for decision-making.
Target Population	Clear description of target population and subgroups analyzed.	Testing reveals heterogeneity and creates multiple subgroups & treatment pathways which may challenge specification of target population groups. Identifying the exact place of a test within care pathway is critical.	Specification of target populations groups according testing rules will guide the selection of relevant comparator and may reduce variability of evaluation findings.
Comparators	Standard care being most widely used.	Multiple potential test designs may exist and makes defining testing interventions a challenge. The sequence of testing and the inclusion of a “no test” comparator is often variable and can lead to different coverage recommendations.	An additional comparison should be considered by splitting the SM treatment. A comparison of the “test first with the new compound/drug” vs. “treat all with new compound/drug” vs. “standard care” is crucial for payers.
Measuring Effectiveness	Systematic review; incorporate real-world factors that modify effectiveness which also may include indirect comparisons.	Estimates of effectiveness relies on various data sources and is more sensitive to adherence and compliance effects.	Strict recommendations that compliance and adherence must be accounted for in sensitivity analysis.
Valuing outcomes	Use appropriate preference-based measures to value differences between the intervention and alternatives (e.g., OALY).	Standard measures (e.g., QALY) have limited applicability and are focused on average population rather than individual/sub-population outcomes. Yet alternative metrics (e.g., personal utility) are underdeveloped and alternative approaches (e.g., cost-benefit analysis) are underused.	Recommendation to incorporate local utilization patterns to improve behavioral assumptions. Further research is needed for quantifying non-health outcomes in evaluations.
Costs and resource use	Measure and value resources that are relevant to study perspective.	Establishing and projecting the additional costs due to testing is challenging.	National price lists of diagnostic test (unit) costs would help avoid reporting variations in costs.
Modeling		Inclusion of sensitivity/specificity and especially false- negative and false- positive considerations will increase structural complexity to establish the relationship between test results and treatment changes and outcomes.	An iterative approach to evaluation is recommended (via early modeling) to identify the need for further evidence generation in alignment with HTA requirements.
Uncertainty	Sensitivity analyses	Extra sensitivity analyses are required for sensitivity/specificity and cost of the test.	Scenario analyses may be more important in SM; especially when considering test characteristics and potential evidence gaps.

## Perspective and target audience

Health Economic evaluations can be performed from the perspective of the society and the national third party payer according to country-specific economic guidelines in health technology assessments. From a methodological point of view, the societal perspective should be preferred over the national third party payer perspective, especially for SM, which requires a more system wide (holistic) approach to perceive the full health- and economic- value taking into considerations costs and long-term benefits having less adverse therapies target toward those who benefit most. However, in practice most economic analyses of SM interventions are performed from a third party perspective, since there is no longitudinal accounting in many healthcare systems in EU and the US which would enable payers to capture long-term cost savings from near-term testing. In addition, pharmaceuticals and diagnostics are considered under separate appraisal and payment processes in many healthcare systems. Only NICE (UK) has so far established a Diagnostic Assessment program (DAP) which carries out cost-effectiveness assessments of selected diagnostics (Bücheler et al., 2014). Funding silos may lead to different payer perspectives, e.g., those who pay for drugs vs. those pay for diagnostic requiring different questions. Hence, the defined perspective which determines the relevant cost and benefits relates much to the discussion on the target audience. For instance, in hospital setting, diagnostic testing is covered by the fee-based DRG system in several EU countries (e.g., Germany, France) or on budget-based systems (e.g., UK, Spain) where a global budget is allocated to local budget holders for payment processes. Further specification of what defines a third party payer, a clear understanding of target audience and broadening to societal perspectives will increase relevance of policy decision making and is useful to identify their evidence needs and incentives to adopt a new technology when proven valuable.

## Target population and comparators

SM interventions may accelerate the evolution and development of clinical treatment pathways which makes the specification of target populations groups a challenge. Technological advances in genetic sequencing and identification of biomarkers have made it feasible to test multiple biomarkers to inform treatment choices, or use algorithms to target screening interval strategies. Also next generation sequencing and whole genome or exome sequencing may allow identifying mutations in multiple genes for multiple conditions in parallel. As a consequence, the number of pathways to include into a model-based economic evaluation may grow exponentially with the number of biomarkers used for stratification (Rogowski et al., 2014). A recent evaluation of a gene recurrence score assay enumerated 1000 potential clinical strategies from 24 clinical testing pathways and 12 unique risk categories based on two tests with two chemotherapeutic regimes (Paulden et al., 2011). In this context it is important to consider that targeted subgroup-specific treatment strategies are clinically plausible and implementable. Identifying the exact place of a SM test within care pathways is crucial and may change the cost-effectiveness outcomes of the intervention (e.g., different results of HER2 testing of trastuzumab in breast cancer patients with adjuvant vs. metastatic settings). This will guide the selection of a relevant comparator - which is usually current standard care in economic evaluations conducted for HTA's -, and determines the appropriate clinical testing strategies to be modeled. Unlike traditional interventions, SM interventions should have at least two comparators: comparisons of the “test first with the new compound/drug” vs. “treat all with new compound/drug” and vs. “standard of care” are recommended although various published cost-effectiveness studies to date have used only the “treat all” strategy as a comparator and ignored the “standard of care” (treat-none with new drug) option. From payers' perspectives, comparisons of the SM approach with “standard care” is often crucial (Merlin et al., 2013). For example, a cost-effectiveness analysis of KRAS testing with cetuximab in colorectal cancer performed by Shiroiwas et al in Japan considered three treatment strategies and outlined that the test-first strategy with cetuximab was dominant vs treat-all-with cetuximab but perhaps not cost-effective vs. the treat-none-with cetuximab strategy (Shiroiwa et al., 2010).

## Measuring effectiveness and outcomes

There is general acknowledgement that the quality of effectiveness data for SM interventions is often weak and challenging to incorporate into standard health economic analyses (Goddard et al., 2012). Effectiveness of SM intervention is a function of both the efficacy of drug and the accuracy of the test and includes considerations on false-positive and false-negative outcomes of testing. One reason why there are relatively few assessments of economic value is that many diagnostic tests do not have widely accepted evidence of clinical utility, i.e., linking test use to patient outcomes. The issues surrounding the definition and measurement of clinical utility are major areas of debate for all kinds of diagnostic testing technologies. Currently, regulators do not require proof of clinical efficacy for a test or even sensitivity/specificity specification which could be used to estimate model effectiveness. Furthermore, data on the effectiveness of laboratory-developed tests is often even more limited due to the ad hoc nature of their development (Faulkner et al., 2012).

There are differences in the evidence generation for the SM-development scenarios. For a test developed in association with a drug (co-development), the economic analysis might be based on randomized controlled trials (RCTs), where the diagnostic test was included in the clinical studies of the drug's efficacy; i.e., sensitivity/specificity data of the test as well as efficacy data of the drug/diagnostic combination are included in the overall outcomes of the trial, which can produce direct evidence of the clinical utility of the test. For a stand-alone test, this is much harder to achieve as RCT's are often not feasible because of ethical reasons, shift to multi-therapeutic regimes, and lack of resources or small patient populations. Real-life data generation is increasingly needed in this case perhaps via prospective cohort studies, observational studies or chart review, as payers might seek additional post-market evidence for clinical utility. It is becoming increasingly apparent that new methods will have to evolve to ensure efficient evidence generation reflecting realistic expectations around evidence standards (thresholds) aligned between stakeholders given the pace of genomic discovery and the associated costs. This implies that health economists and decision makers must be prepared to accept data that have come from different settings (case-control and observational) outside RCT's. Potential alternative solutions may involve the use of novel trial designs, such as adaptive clinical trials.

Furthermore it is to consider, that the overall effectiveness of the SM intervention doesn't only rely on the development of new treatment modalities, but also on providers and patients behavior when using diagnostic-based therapies. How patients are managed in practice is important and will influence the adoption of new technologies (e.g., examples warfarin PGx testing and TMPT testing for patient taking 6-mercaptopurine or allopurinol). SM underscores the need for additional information on patients and physicians response to diagnosis and will require post-approval data collection. Accounting for compliance and adherence (e.g., by use of local utilization pattern to improve behavioral assumptions) will reduce variability of findings and should be incorporated into sensitivity analyses. The recently drafted guidelines for preparing assessment reports for the Medical Services Advisory Committee- Service Type: Investigative (version 1.3) in Australia specifically request a supplementary analysis of the non-health related impacts of diagnostic testing^3^.

The impact of an intervention on health status (e.g., cost per QALY's or life year saved) is the preferred outcome measure for several EU governmental advisory bodies (e.g., NICE, SMC, TLV, or CRM) as recommended in the health economic guidelines. However, for third party payers such standard measures may have limited applicability in assessing SM interventions rather requesting cost-offsets and budget impact information to address affordability issues in various health care systems. Methodological issues regarding the valuation of health outcomes for SM, particular the quality-adjustment of utility component in QALYs, are similar to those faced by other health care intervention. There is an ongoing discussion in academia how standard value assessment metrics can be expanded by personal utility data, as current metrics is focussed on average population based preferences rather than individual patient preference valuation. Capturing information on personal utility may be important, because additional benefits may arise from a patient's increased certainty about the likelihood of successful treatment—the “value of knowing” (although ultimately always to be aggregated to population levels). This might affect adherence and thereby patient outcomes. Yet, alternative metrics (e.g., personal utility) are underdeveloped or alternative approaches underused (e.g., state of choice, willingness to pay) in policy decision making (Buchanan et al., 2013). Further, research in this area is required to provide guidance for quantifying and incorporating non-health outcomes in economic evaluations.

## Estimate resource use and cost

The costing methodology is straightforward and there may not be methodological differences with the costing methodology in health economic evaluations for traditional pharmaceuticals. Cost calculations in economic evaluation require total average costs (including capital and allocated overhead costs) derived from resources consumed and unit cost measures based on economic (opportunity costs; Conti et al., 2010) Yet, establishing and projecting the additional costs due to testing may provide challenges for analysts. A broad range of direct testing costs may include additional clinic visits, sample collection and testing, the cost of subsequent treatment and genetic counseling as well as re-testing considerations. However, the complete estimation of costs relates to the type of cost items and primarily not a methods issue, beyond the perspective chosen.

Often, there are challenges to identify the unit cost of tests which may depend on number of tests performed or be part of platform diagnostics with multiple applications. Unlike pharmaceutical, there is no national list of available genomic or other tests, as often each laboratory is free to set their own price (or charge) to clinicians requesting the test and negotiations between suppliers and users often occur at local levels. Large variation in the unit cost of these tests can affect the findings of an economic evaluation and increase uncertainty in the estimated relative cost-effectiveness of a test. Sensitivity analysis should address robust cost estimates relevant to diagnostic testing, yet national price lists of diagnostic test costs would help avoid the currently reported variation in costs (NHS-UK Genetic Testing Network, 2011). One costing question is related to the perspective. If we assume that a test is performed in an inpatient setting, then from a payer perspective, only the diagnosed related group (DRG), including all inpatient resource utilization, needs to be applied and the hospital must take care of being able to finance the test within the DRG. However, from a societal perspective, the cost of the test should be added to the DRG assuming that the current DRG reflects an opportunity cost to the hospitalization. Therefore, micro-costing approach would be most appropriate in order to capture the real/true costs.

## Modeling and dealing with uncertainty

The existing modeling techniques are appropriate and can be applied for cost-effectiveness models in SM, given that special issues are taken into consideration. The inclusion of sensitivity/specificity and especially false negatives and false positives, requires additional structural complexity in order to make the link between the test and the medication and the subsequent clinical and economic outcomes. Another issue is dealing with gaps in the evidence base, especially for stand-alone tests. Information on treatment patterns, its costs and outcomes, are often lacking, especially for false positive and false negative patients. There is a need to identify best practices for economic modeling including approaches which address these evidence gaps in a manner that is both acceptable to payers and feasible for test manufactures. Thus, extrapolation methods are required in order to extrapolate the short-term sensitivity/specificity data to long-term economic outcomes, as shown in a recent paper by Fugel and Nuijten (2014) Given that health economists will increasingly be faced with poor quality effectiveness and cost data early modeling approaches will become more common in early development stages to better understand the HE value of new technologies. An iterative approach could then be employed that systematically and explicitly considers the need for further evidence to reduce decision uncertainty, and is consistent with an approach to HTAs known as constructive technology assessment (Sculpher et al., 1997; Shabaruddin et al., 2015).

Sensitivity analyses aim at providing information on the degree of uncertainty in economic evaluations and it is currently the most widely applied method of dealing with uncertainty in economic evaluations (Critchfield et al., 1986). Because of the more complex structure, lack of data, and extrapolation, the uncertainty level in the SM model is higher than in a comparable model for traditional pharmaceuticals. In addition, the efficacy of the stand-alone test is often based on a small sample size leading to extra uncertainty, thus, extra sensitivity analyses are required for sensitivity/specificity and cost of the test. In SM, there are more gaps in information and the number of possible assumptions increases with the number of parameters added which may cause interpretation problems. A practical way to overcome this problem is the use of scenarios, in which several factors are set to reflect a specific situation, such as the best-case and worst-case scenarios (Vegter et al., 2008). Hence, for the SM approach, scenario analyses may be more important than sensitivity analyses, especially when considering test characteristics and potential evidence gaps, because there are rather issues on the quality of the data than the distribution of the variable. The structural uncertainty of the assumptions due to gaps in data is larger than the uncertainty due to statistical distribution. Specific scenario analyses in SM, which are not relevant in traditional pharmaceuticals, may be required for a range of estimates in turn, but it may also be possible to perform a “multi-scenario” analysis, where the effect of simultaneous changes in different assumptions is examined on the outcomes of the study.

A probabilistic sensitivity analysis (PSA) permits the analyst to assign a range and distribution to input variables (Doubilet et al., 1985). The results of a PSA are presented in a cost-effectiveness acceptability curve, which displays the probability that a new treatment is the most cost-effective treatment considered in the analysis at a range of different threshold ICER values representing what society might be willing to pay to gain one e.g., QALY. However, the results of a PSA for SM may need to be considered with more prudence than with traditional pharmaceuticals. Gaps in information and subsequent assumptions cannot be captured by a statistical distribution and therefore this type of uncertainty cannot be included fully in a PSA.

## Conclusion and future research

In general, we can conclude that current health economics methodology can be applied for SM, although various aspects of the guidelines require specific attention for stratified medicine approaches. These aspects comprise considerations on the choice of comparator, measuring effectiveness and outcomes, appropriate modeling structure and the scope of sensitivity analyses. Many of these aspects refer to a lack of evidence on testing heterogeneity and the quality of effectiveness data. Notably, the level of economic evidence for SM interventions may differ from what is generally experienced with traditional pharmaceuticals, thus stressing the need to identify best practice for economic modeling including approaches which address evidence gaps in a manner that is both acceptable for payers and feasible for test manufactures. This may involve the use of novel trial designs, such as adaptive clinical trials, evidence from observational studies, and the use of coverage-with-evidence development and real-world evidence collection for both drugs and diagnostics. However, the evaluations of both test-treatment interventions (companion diagnostic) and stand-alone diagnostics is occurring in a complex legal, regulatory and reimbursement environment which does not currently fit with SM approaches. New incentive structures are needed to increase the efficiency of evidence generation. Previous suggestions for economic incentives for evidence generation include value-based price flexibility, intellectual property protection from evidence generated and public investment to complement the effort of payers and manufactures (Towse and Garrison, 2013).

SM underscores the need for additional information on patients and physicians response to diagnosis which is not readily available from clinical trials or administrative data sets. Accounting for compliance and adherence (e.g., by the use of local utilization pattern to improve behavioral assumptions) will provide insight into variability of findings and should be incorporated into sensitivity analyses. Health economist may need to take new accountabilities when using observational research methods to perform additional value from utilization data to payers.

Incorporating complex genetic or genomic data into cost-effectiveness analyses is a challenge that will grow as next generation sequencing technologies enter clinical practice. While there is no need to develop completely new tools, there are requirements for some refinement by including sensitivity and specificity consideration of the test as well as to address consequences of false-negative and false- positive test results on the value proposition. This may require further methodology development to address the increased complexity and the need for additional analyses associated with the testing component. Further research should also consider examining other approaches to measuring values for SM interventions. The specific aspects outlined in this article suggest there may be opportunities to improve current guidelines for economic evaluation of SM interventions.

## Author contributions

HF initiated this methodology review, addressed issues through literature review, informal interviews and discussions with international experts and prepared final manuscript. MN participated in interviewing international experts, supported the results generation and provided critical comments to the final manuscript. MP and KR provided substantial commentary for the optimization of this methodology review and critically revised it for important intellectual content.

### Conflict of interest statement

The authors declare that the research was conducted in the absence of any commercial or financial relationships that could be construed as a potential conflict of interest.

## Footnotes

^1^Pharmacoeconomic Guidelines Around The World, www.Ispor.org/PEguidelines/index.asp

^2^National Institute for Health and Clinical Excellence. The guideline manual—assessing cost effectiveness, https://www.nice.org.uk/guidelinemanual/7-assessing-cost-effective

^3^www.health.gov.au/internet/hta/publishing.nsf - technical guidelines for preparing assessments reports for the Medical Services Advisory Committee - Service Type: Investigative (version 1.3). Draft for public consultation 2015; www.pbs.gov.au/pbac-guidelines/product type 4- hybrid technologies and co-dependent technologies, Version 5.0, March 2016
